# Cognitive dysfunction and migraine

**DOI:** 10.1186/s10194-018-0933-4

**Published:** 2018-11-15

**Authors:** Doga Vuralli, Cenk Ayata, Hayrunnisa Bolay

**Affiliations:** 1 Department of Neurology and Algology, Gazi University Faculty of Medicine, Besevler, 06510 Ankara, Turkey; 2 Neuropsychiatry Center, Gazi University, Besevler, 06510 Ankara, Turkey; 3 Neurovascular Research Lab, Department of Radiology, Massachusetts General Hospital, Harvard Medical School, Charlestown, MA USA; 4 Stroke Service, Department of Neurology, Massachusetts General Hospital, Harvard Medical School, Charlestown, MA USA

**Keywords:** Migraine, Cognitive dysfunction, Clinical studies, Neuropsychological tests, Neurophysiology, Neuroimaging

## Abstract

Cognitive dysfunction has recently gained attention as a significant problem among migraine sufferers. All of the clinical studies show poor cognitive performance during migraine attacks, though, the interictal data are conflicting. Migraineurs show impaired cognitive function interictally in most of the clinic-based studies. Population-based studies did not reveal a difference in cognitive functions between migraineurs and controls. The specific cognitive domains involved are information processing speed, basic attention, executive functions, verbal and non-verbal memory and verbal skills. Neurophysiological, imaging and pharmacological studies support clinical symptoms of cognitive impairment in migraine. Longitudinal studies do not suggest progressive cognitive decline over time in migraine patients. Preventive medications and comorbid disorders such as depression and anxiety can impact cognitive function, but cannot fully explain the cognitive impairment in migraine. In contrast to migraine, tension type or cluster headache are not associated with cognitive impairment, at least during headache-free periods.

## Background

Migraine is a common headache disorder with an average prevalence of 20,2% in women and 9,4% in men [1–3]. Migraine headache is the third most prevalent disorder and the sixth highest specific cause of disability worldwide [4].

Subjective cognitive decline is not uncommon in migraine patients. Although cognitive symptoms are not considered among the core symptomatology of migraine, many migraineurs often complain of intellectual impairment, particularly deficits in attention and memory. Cognitive symptoms are frequent in the premonitory phase and headache phase of a migraine attack and may also persist in the postdrome. Some migraineurs also complain of cognitive symptoms outside migraine attacks. Acute attack treatments are not always successful at relieving cognitive symptoms. Cognitive dysfunction, particularly impairment in executive function, also contributes to migraine attack-related disability. Indeed, cognitive symptoms ranked second after pain in intensity and attack-related disability [5], making this a relevant target in migraine attack management. High levels of attack related disability are also related to decreased cognitive performance [6]. Worse pain severity, higher levels of depression and anxiety, and poor sleep quality and decreased sleep duration, are all associated with more severe subjective cognitive decline [7].

## Methods

Studies for our qualitative review were selected from PubMed and PsychInfo databases using the key words “Migraine”, “Headache”, “Cognition”, “Cognitive Functions” and “Cognitive Impairment” and reference sections of these studies provided further studies that evaluated cognitive functions on migraine patients. This search was performed to establish the results of neuropsychological assessment for monitoring cognitive functions in migraine patients. Studies were included 1) If they used *validated neuropsychological* and *cognitive* assessments. Studies were excluded 1) If they included patients with other neurologic disorders aside from migraine, 2) If migraine was not differentiated from other primary headaches, 3) If they did not have a control group and 3) If they were not published in English. In addition, neuroimaging, neurophysiological and pharmacological studies were also reviewed.

No randomized controlled trial was found in the literature regarding cognitive functions in migraine. Case control studies, population based prospective cohort studies and case series were found in the literature and case control studies and population based prospective cohort studies were included in the review.

### Overview of clinical studies on cognitive dysfunction in migraine

Concordant with the subjective complaints, all objective studies of cognitive performance in migraineurs consistently show various degrees of impairment during an attack [8–12]. Even though interictal studies show conflicting results [13–19], most of the clinic-based studies reported worse cognitive performance interictally in migraineurs compared to healthy controls. Clinic-based studies, evaluated cognitive functions in migraine had small sample sizes and enrolled patients with higher frequency of attacks and higher headache intensity than migraine patients in the general population. Moreover patients from clinics usually have more severe accompanying conditions such as anxiety and depression. Disease severity measures such as disease duration, frequency and duration of headache attacks and pain intensity may be a factor in cognitive impairment in migraine patients. Indeed, Huang et al. [13] showed that the increased frequency and the longer durations of migraine attacks are correlated with worse cognitive function. There are limited clinic based studies which found no difference between migraine patients during interictal period and healthy controls regarding cognitive functions. In a clinic based functional magnetic resonance imaging (fMRI) study [20], 14 migraine without aura and 14 migraine with aura patients and 14 healthy controls underwent a resting state with high field fMRI examination and cognitive evaluation with a battery of standardized neuropsychological tests. Even though no abnormalities were found in neuropsychological evaluation of migraine patients, an alteration in functional connectivity were shown in migraine with aura patients compared to migraine without aura patients and healthy controls. In another clinic based study, response time in word priming, orientation search and temporal discrimination tasks were comparable among 12 migraine with aura, 12 migraine without aura and 12 controls [19]. In the latter study, some patients were under migraine prophylaxis and headache frequency of the migraine patients were lower compared to other clinical based studies.

Population-based studies have the advantages of large size and generalizability. Most population-based studies reported no difference between migraine patients and subjects without headache. In one cross-sectional, population based study performed in 99 migraineurs and 1768 people without migraine, migraine patients did not show significantly lower cognitive performance [21]. However, in this study migraine diagnosis was based on self-reporting. In another cross-sectional, population based study performed in 1393 twins, 536 with migraine diagnosed in neurology departments (347 migraine without aura and 157 migraine with aura), the mean cognitive scores on fluency, digit span, delayed word recall, and symbol digit substitution test were comparable between patients with migraine or one of the migraine subtypes and non-migraineurs [22]. In fact, one population-based study of middle aged and elderly migraineurs showed even better cognition than non-migraineurs according to Mini Mental State Examination and global cognition assessed by a general cognitive factor [23]. In this study, the cognitive abilities of migraine with aura patients and those with a past history of migraine were markedly better than migraine patients without aura and those with ongoing attacks. Even though the study had strengths such as being population-based, large number of participants and detailed cognitive evaluation, it also had limitations such as older age group and the retrospective nature of migraine diagnosis. The relatively better cognitive status could be related not to migraine itself but to other confounders such as lifestyle changes (e.g. less alcohol intake, avoiding triggers such as dehydration, fasting, lack of sleep and smoking) and medications used. In fact, age, alcohol intake, smoking, presence of type 2 diabetes and diastolic blood pressure were lower, and anti-hypertensive usage and female/male ratio were higher in the definite and probable migraine groups in this study [23]. In contrast to above studies, there are two population based studies which found impairment in cognitive functions in migraine patients. In the first one with 61 migraine patients, 50 non-migraine headache patients and 367 headache-free controls [24], migraine sufferers performed worse in a task of sustained attention and processing speed, which are related to the activation and integrity of prefrontal cortex, but not in the verbal fluency tasks, working memory (digit span backwards), inhibitory control (Stroop test), or measures of verbal and visual learning and recall. The other one revealed significantly poorer performance in Trail Making Test version B in migraine patients, suggesting an impairment in executive functions, processing speed and attention [25].

Longitudinal study design has the advantage of showing the association between migraine and cognitive decline over time. Longitudinal studies assessing cognitive function in migraine have used population-based samples. Longitudinal studies did not provide any evidence for cognitive decline over time in migraine patients. In most studies, migraine was diagnosed using a questionnaire based on International Classification of Headache Disorders-I (ICHD-I) or ICHD-II [26, 27], or based on patients’ self-reports [28, 29]. Participants were assessed at least one more time after the baseline evaluation and average follow-up ranged between 3.4 to 23 years [26–29]. These studies showed that patients who experience migraine with aura or migraine without aura were not at increased risk of cognitive decline, and in some cognitive tests migraine patients showed less decline over time. For example, in Wechsler test in the Epidemiology of Vascular Aging Study (EVA), and in tests of immediate and delayed recall in the Baltimore Epidemiologic Catchment Area study, migraineurs experienced slower decline over time than those without migraine [26, 27]. However, in these four longitudinal population based studies, the main aim was not to compare cognitive functions between migraineurs and controls without headache and comparisons were done as a part of larger studies. In Maastricht Aging Study, the aim was to define the determinants of cognitive aging in which migraine was one of the reported medical conditions by the patients. The EVA study was a longitudinal study of vascular and cognitive ageing in a population-based cohort, migraine was assessed in the third wave of the study and cognitive assessments from the third wave were used as the baseline measurements. Baltimore Epidemiologic Catchment Area study was actually a longitudinal population-based study that mainly aimed to evaluate the prevalence and incidence of Diagnostic and Statistical Manual of Mental Disorders (DSM) mental disorders in five regions of the United States. The data from the third and fourth waves of the Baltimore Epidemiologic Catchment Area study was reported [26] separately, as an interview for migraine diagnosis took place in the third wave. The Women’s Health Study was a randomised, placebo controlled clinical trial that investigated the role of low dose aspirin and vitamin E in the prevention of cardiovascular disease and cancer. Rist and colleagues published the cognitive sub cohort data from Women’s Health Study and included subjects who provided information about their migraine status and participated in cognitive testing during follow-up [28].

In another longitudinal study, health and behavior of individuals born in Dunedin, New Zealand between 1 April 1972 and 31 March 1973 were investigated and cognitive, neuropsychological and medical assessments were done at ages 3, 5, 7,9, 11, 13, 15, 18, 21 and 26 [30]. At age 26, individuals were assessed for migraine and retrospectively 114 migraine patients, 109 tension type headache (TTH) patients and 739 controls without headache were compared for performance in cognitive and neuropsychological tests. Migraine patients had impaired verbal ability (especially language reception) at ages 3, 7, 9, 11, and 13, before the development of headache attacks, compared to controls without headache or subjects with tension type headache, but no decline with age was observed, suggesting that migraine did not cause verbal impairment itself but they had a shared risk factor [30]. Although longitudinal studies do not provide convincing evidence that cognitive function worsens over time in migraine patients, participants in most longitudinal studies were older than those in cross sectional and clinic-based studies [8–10, 13–15, 31–37], and both active (< 1 year since last attack) and non-active migraine patients were included (> 1 year since last attack), as potential confounders. The studies evaluating cognitive functions in migraine patients categorized according to age groups are summarized in Tables 1, 2, 3.

**Table 1:** Studies evaluating cognitive function in children with migraine

Migraine Phase	Cognitive Domains Affected in Migraine	Study design	Evaluation Methods	Outcome	Reference
Interictal	Executive functions	Cross sectional, clinic based	Trail Making Test, Stroop Test	Impairment in executive functions in children with migraine compared to healthy controls	Costa-Silva et al., 2016 [31]
Interictal	Attention	Cross sectional, clinic based	Trail Making Test, Test of Visual Attention	Migraine group had difficulties in selective attention and mainly in alternate attention	Villa et al., 2009 [32]
Interictal		Cross sectional, clinic based	WISC-R	Impairment in attention in children with migraine	Moutran et al., 2011 [48]
Interictal		Cross sectional, clinic based	Trail Making Test	Impairment in selective and divided attention in children with migraine compared to control subjects	Costa-Silva et al., 2016 [31]
Interictal	Visuospatial memory	Cross sectional, clinic based	Rey Figure Test (Delayed recall)	Delayed recall is impaired in children with migraine compared to controls	D’Andrea et al., 1989 [33]
Interictal		Cross sectional, clinic based	WISC-R	Impairment in visuospatial memory in children with migraine	Moutran et al., 2011 [48]
Interictal	Verbal memory	Cross sectional, clinic based	Logical Memory Test, Ten Word Learning Test	Memory is impaired in children with migraine compared to non-migrainous children	D’Andrea et al., 1989 [33]
Interictal		Cross sectional, clinic based	WISC-R	Children with migraine performed worse than healthy controls	Parisi et al., 2009 [34]
Interictal		Cross sectional, clinic based	WISC-R	Children with migraine had worse performance in verbal memory	Moutran et al., 2011 [48]
Interictal		Cross sectional, clinic based	Rey Auditory Verbal Learning Test	Children with migraine perfomed significantly worse than control subjects	Costa-Silva [31] et al., 2016
Interictal	Information processing speed		Simple Visual Reaction Time Task	A significant dysfunction was observed in the information processing rate in children with migraine	Riva et al., 2006 [35]
Interictal		Cross sectional, clinic based	WISC-R	Children with migraine had decreased processing speed	Moutran et al., 2011 [48]
Interictal		Cross sectional, clinic based	Trail Making Test	Children with migraine had slower information processing speed than controls	Costa-Silva et al., 2016 [31]
Interictal	Verbal skills	Longitudinal, population based	WISC-R	Migraine patients were significantly impaired on tests of verbal ability between the ages 3 and 13 however no decline with age was observed	Waldie et al., 2002 [30]
Interictal		Cross sectional, clinic based	WISC-R	Children with migraine had impaired verbal skills compared to healthy controls	Parisi et al., 2009 [34]
Interictal		Cross sectional, clinic based	Verbal Fluency Test	Children with migraine displayed lower performance in the animals category	Costa-Silva et al., 2016 [31]
Interictal	Perceptual organization	Cross sectional, clinic based	WISC-R	Perceptual organization was disturbed in children with migraine	Moutran et al., 2011 [48]
Unspecified		Cross sectional, clinic based	WISC-III	Children with migraine had lower perceptual organization competence than children with tension-type headache or healthy controls	Esposito et al., 2012 [36]

*WISC-R* Wechsler Intelligence Scale for Children – Revised, *WISC-III* Wechsler Intelligence Scale for Children Third Edition

**Table 2:** Studies evaluating cognitive function in migraine patients < 50 years

Migraine Phase	Cognitive Domains Affected in Migraine	Study design	Evaluation Methods	Outcome	Reference
Ictal, interictal	Executive functions	Cross sectional, clinic based	HCC-ANAM	Migraine attack negatively affects executive functions	Farmer et al., 2000, 2001 [9, 10]
Ictal		Cross sectional No information about recruitment of migraine sample	MEWT- Matching to Sample	Working memory decline at the onset of a migraine attack	Edwards et al., 2013 [11]
Interictal		Cross sectional, clinic based	Trail Making Test, Paced Auditory Serial Addition Test	Impaired working memory and sustained attention in migraine patients	Zeitlin et al.,1984 [39]
Interictal		Cross sectional, clinic based	WAIS-R Digit Span Subtest	Impairment in sustained attention in migraine patients	Hooker et al., 1986 [14]
Interictal		Cross sectional Migraine patients were recruited from college students	Neuropsychological Evaluation System-1) Continuous Performance with Pictures 2) Continuous Performance with Letters 3)Color Word Task 4)Symbol Digit Substitution Task	Impairment in sustained attention in migraine patients	Mulder et al.,1999 [43]
Interictal		Cross sectional, clinic based	Trail Making Test-B, Wisconsin Card Sorting Test	Impaired executive functions in migraine patients	Camarda et al., 2007 [47]
Interictal		Cross sectional, clinic based	MoCA	Migraineurs performed worse in executive functions part	Huang et al.,2017 [13]
Chronic migraine^a^		Cross sectional, clinic based	Tower of Hanoi-3	Impairments in planning, sequencing skills and working memory in migraine patients	Mongini et al., 2015 [38]
Interictal		Cross sectional, population based	Trail Making Test-B	Poorer performance in Trail Making Test-B in migraine patients suggesting an impairment in executive functions	Pellegrino Baena et al., 2018 [25]
Interictal		Cross sectional Migraine patients were recruited from college students	Trail Making Test	No difference between migraine patients and controls regarding working memory	Burker et al., 1989 [52]
Interictal		Cross sectional The migraine patients were recruited from a meeting of a local organization for migraine patients and from patients of general practitioners	Continuous Performance Test, Digit Span Backwards	No difference between migraine patients and control subjects regarding sustained attention and working memory	Leijdekkers et al., 1990 [16]
Interictal		Cross sectional, clinic based	Trail Making Test, Wisconsin Card Sorting test	No difference between migraine patients and control subjects	Lo Buono et al., 2017 [20]
Ictal, interictal	Attention	Cross sectional, clinic based	HCC-ANAM - Continuous Performance Test	Migraine attack negatively affects attention	Farmer et al., 2000, 2001 [9, 10]
Interictal		Cross sectional, clinic based	Stroop Test, Trail Making Test	Impaired attention in migraine patients	Zeitlin et al., 1984 [39]
Interictal		Cross sectional Migraine patients were recruited from college students	Neuropsychological Evaluation System-1) Continuous Performance with Pictures 2) Continuous Performance with Letters 3) Color Word Task	Migraine patients were slower in tasks requiring selective attention	Mulder et al., 1999 [43]
Interictal		Cross sectional, clinic based	Boston Scanning Test (or Visual Continuous Performance Test)	Impaired attention in migraine patients	Le Pira et al., 2000 [15]
Interictal		Cross sectional, population based	Trail Making Test-B	Impairment in attention in migraine patients	Pellegrino Baena et al., 2018 [25]
Interictal		Cross sectional Migraine patients were recruited from college students	Trail Making Test	No difference between migraine patients and controls	Burker et al., 1989 [52]
Interictal		Cross sectional No information about recruitment of migraine sample	Automatic Attention Task and Conscious Visual Attention Task	High visual discomfort, rather than migraine is associated with poorer performance	Conlon et al., 2001 [18]
Ictal, interictal	Visuospatial memory	Cross sectional, clinic based	HCC-ANAM - Matching to sample	Visual memory was negatively affected during migraine attack	Farmer et al., 2000, 2001 [9, 10]
Ictal		Cross sectional No information about recruitment of migraine sample	MEWT- Matching to Sample	Visual memory decline at the onset of a migraine attack	Edwards et al., 2013 [11]
Interictal		Cross sectional, clinic based	Rey Complex Figure test	Impaired visuospatial memory in migraine patients	Le Pira et al., 2000 [15]
Interictal		Cross sectional Migraine patients were recruited from college students	Rey-Osterreith Complex Figure Test	No difference between migraine patients and controls	Burker et al., 1989 [52]
Interictal		Cross sectional The migraine patients were recruited from a meeting of a local organization for migraine patients and from patients of general practitioners	Pattern Memory Test	No significant difference between migraine patients and control subjects	Leijdekkers et al., 1990 [16]
Ictal	Verbal memory	Cross sectional, clinic based	California Verbal Learning Test	Verbal memory performance of migraineurs were decreased during a migraine attack	Gil-Gouveia et al., 2015 [8]
Interictal		Cross sectional, clinic based	WMS-Revised	Verbatim recall of short stories after 30-min delay was performed significantly worse in migraineurs	Hooker et al., 1986 [14]
Interictal		Cross sectional, clinic based	California Verbal Learning Test	Verbal memory impairment in migraine patients	Le Pira et al., 2000 [15]
Interictal		Cross sectional, clinic based	MoCA	Migraineurs performed worse in verbal memory	Huang et al.,2017 [13]
Interictal		Cross sectional, clinic based	Memory for Intentions Screening Test	Migraine without aura patients performed significantly worse than healthy controls on time-based and event-based scales	Santangelo et al. 2018 [37]
Interictal		Cross sectional Migraine patients were recruited from college students	Selective Reminding Test	No difference between migraine patients and controls	Burker et al., 1989 [52]
Interictal		Cross sectional The migraine patients were recruited from a meeting of a local organization for migraine patients and from patients of general practitioners	Associate Learning	No significant difference between migraine patients and control subjects	Leijdekkers et al., 1990 [16]
Interictal		Cross sectional, clinic based	Rey Auditory Verbal Learning Test	No difference between migraine patients and healthy controls	Lo Buono et al., 2017 [20]
Interictal	Recognition memory	Cross sectional, clinic based	National hospital forced choice recognition test for words and faces	Migraine patients had worse performance in recognition memory tasks	Zeitlin et al., 1984 [39]
Interictal		Cross sectional, clinic based	Memory for Intentions Screening Test	Migraine without aura patients performed significantly worse than healthy controls on recognition subscale	Santangelo et al. 2018 [37]
Ictal, interictal	Information processing speed	Cross sectional, clinic based	HCC-ANAM - Simple reaction time	Increased cognitive processing time during a migraine attack	Farmer et al., 2000, 2001 [9, 10]
Ictal		Cross sectional No information about the recruitment of migraine sample	MEWT-1) Simple reaction time 2) Procedural reaction time	Impairment in mental processing in migraine patients during a migraine attack	Edwards et al., 2013 [11]
Ictal		Cross sectional, clinic based	The word reading task of the Stroop	Reduced processing speed in migraine patients during a migraine attack	Gil-Gouveia et al., 2015 [8]
Post-ictal		Cross sectional No information about the recruitment of migraine sample	Computerized posner paradigm, calculating visual reaction times	Reaction times (RTs) during the post-ictal period were significantly higher than the RTs during interictal period	Mazzucchi et al., 1988 [12]
Interictal		Cross sectional, clinic based	Stroop Test, trail making test, choice reaction time, paced auditory serial addition test	Increased reaction time in migraine patients	Zeitlin et al., 1984 [39]
Interictal		Cross sectional, clinic based	WAIS-R Digit Span Subtest	Reduced visuomotor processing speed in migraine patients	Hooker et al., 1986 [14]
Interictal		Cross sectional, clinic based	Digit Symbol Substitution Test	A poorer performance in visuomotor processing speed in migraine patients	Calandre et al., 2002 [46]
Interictal		Cross sectional No information about recruitment of migraine sample	Automatic attention task and conscious visual attention task	High visual discomfort, rather than migraine is associated with poorer performance	Conlon et al., 2001 [18]
Interictal		Cross sectional, population based	Trail Making Test-B	Decreased processing speed in migraine patients	Pellegrino Baena et al., 2018 [25]
Interictal		Cross sectional No information about the recruitment of migraine sample	Computerized posner paradigm, calculating visual reaction times	Migraine patients had RTs during the interictal period that were not significantly different from control subjects	Mazzucchi et al., 1988 [12]
Interictal		Cross sectional The migraine patients were recruited from a meeting of a local organization for migraine patients and from patients of general practitioners	Simple Reaction Time, Symbol Digit Substitution Task, Color Word Vigilance Test, Pattern Comparison Task, Sternberg Memory Scanning Task	No significant difference between migraine patients and control subjects	Leijdekkers et al., 1990 [16]
Interictal		Cross sectional, clinic based	Word Priming Task, Orientation Search Task, Temporal Discrimination Task	No difference between migraine patients and control subjects regarding response time	Palmer et al., 1998 [19]
Interictal		Cross sectional, clinic based	Trail Making Test-Part A	No difference between migraine patients and healthy controls	Lo Buono et al., 2017 [20]
Ictal	Verbal skills	Cross sectional, clinic based	The word reading task of the Stroop	Disturbances in reading was observed in migraine patients during a migraine attack	Gil-Gouveia et al., 2015 [8]
Interictal		Cross sectional, clinic based	Mill Hill Vocabulary Scale	No difference between migraine patients and controls	Zeitlin et al., 1984 [39]
Interictal		Longitudinal, population based	WISC-R	Migraine patients were significantly impaired on tests of verbal ability between the ages 3 and 13 however no decline with age was observed	Waldie et al., 2002 [30]
Interictal		Cross sectional Migraine patients were recruited from college students	Aphasia screening test, Speech Sounds Perception Test	No difference between migraine patients and controls	Burker et al., 1989 [52]
Interictal		Cross sectional The migraine patients were recruited from a meeting of a local organization for migraine patients and from patients of general practitioners	Vocabulary Test	No significant difference between migraine patients and control subjects	Leijdekkers et al., 1990 [16]
Interictal		Cross sectional, clinic based	Verbal Fluency Test	No difference between migraine patients and healthy controls	Lo Buono et al., 2017 [20]
Ictal	Calculation	Cross sectional, clinic based	HCC-ANAM - Mathematical processing	Impairment in mathematical processing during a migraine attack	Farmer et al., 2000, 2001 [9, 10]
Interictal		Cross sectional, clinic based	Paced auditory serial addition test	Disturbance in calculation ability in migraine patients	Zeitlin et al., 1984 [39]
Interictal		Cross sectional, clinic based	MoCA	Calculation was impaired in migraine patients	Huang et al.,2017 [13]
Ictal	Somatosensory information processing	Cross sectional, clinic based	STD	Prolonged STD threshold values	Boran et al., 2016 [61] Vuralli et al., 2017 [62]
Chronic migraine (during headache and headache free periods)		Cross sectional, clinic based	STD	Prolonged STD threshold values during both during headache and headache free periods	Vuralli et al., 2016 [63]

^a^It was not stated whether the patients were evaluated on a headache day or a headache free day

*HCC-ANAM* Headache Care Center-Automated Neuropsychological Assessment Metrics, *MEWT* Mental Efficiency Workload Test, *WAIS-R* Wechsler Adult Intelligence Scale-Revised, *WMS* Wechsler Memory Scale, *STD* Somatosensory temporal discrimination

**Table 3:** Studies evaluating cognitive function in migraine patients > 50 years

Migraine Phase	Cognitive Domains Affected in Migraine	Study design	Evaluation Methods	Outcome	Reference
Interictal	Executive functions	Cross sectional Subjects were recruited from primary health care centers	Digit Span Backwards, Stroop test	No significant difference between migraine patients and control subjects without headache	Martins et al., 2012 [24]
Unspecified		Cross sectional, population based	Letter Digit Substitution Test	No difference between migraine patients and control subjects regarding sustained attention and mental flexibility	Jelicic et al. 2000 [21]
Unspecified		Cross sectional, population based	Fluency Test, Symbol Digit Substitution Task, Digit Span Test	No difference between migraineurs and non-migraineurs	Gaist et al., 2005 [22]
Unspecified^a^		Cross sectional, population based	Stroop Test, Verbal Fluency Test	Migraine patients performed better than participants without migraine	Wen et al., 2016 [23]
Unspecified^b^		Longitudinal, population based	Stroop Test, Letter Digit Substitution Test, MMSE	No significant difference between migraine patients and controls	Baars et al., 2010 [29]
Unspecified^c^		Longitudinal, population based	Digit Symbol Substitution Test, Trail Making Test, MMSE, Word Fluency Test	No significant difference between migraine patients and controls	Rist et al., 2011 [27]
Unspecified^d^		Longitudinal, population based	Category Fluency Task, Telephone Interview for Cognitive Status (Telephone adaptation of MMSE)	No significant difference between women with migraine or a past history of migraine and women without migraine	Rist et al., 2012 [28]
Interictal	Attention	Cross sectional Subjects were recruited from primary health care centers	Symbol Search	Migraine patients performed worse on the task of attention	Martins et al., 2012 [24]
Interictal	Visuospatial memory	Cross sectional Subjects were recruited from primary health care centers	WMS-III Delayed Visual Memory, WMS-III Memory for Faces, Famous FacesTest.	No difference between migraine patients and controls without headache	Martins et al., 2012 [24]
Unspecified^c^		Longitudinal, population based	Raven Progressive Matrics, Benton Visual Retention Test	No significant difference between migraine patients and controls	Rist et al., 2011 [27]
Interictal	Verbal memory	Cross sectional Subjects were recruited from primary health care centers	California Verbal Learning Test, Wechsler Abbreviated Scale of Intelligence Vocabulary Subtest	No difference between migraine patients and controls without headache	Martins et al., 2012 [24]
Unspecified		Cross sectional, population based	Verbal learning test	Migraine had no influence on memory in young/middle-aged or older adults	Jelicic et al. 2000 [21]
Unspecified		Cross sectional, population based	Delayed Word Recall Test	No difference between migraineurs and non-migraineurs	Gaist et al., 2005 [22]
Unspecified^a^		Cross sectional, population based	15-Word Learning Test	No difference between migraine patients and controls	Wen et al., 2016 [23]
Unspecified^b^		Longitudinal, population based	Visual Verbal Learning Test, MMSE	No difference between migraine patients and controls	Baars et al., 2010 [29]
Unspecified^e^		Longitudinal, population based	Modified version of Rey Verbal Learning Test, MMSE	Migraineurs showed significantly less decline over time on the tests of immediate and delayed recall compared to non-migraineurs	Kalaydjian et al., 2007 [26]
Unspecified^c^		Longitudinal, population based	Rey 15-word Memory Test, Raven Progressive Matrics	No significant difference between migraine patients and controls	Rist et al., 2011 [27]
Unspecified^d^		Longitudinal, population based	East Boston Memory Test, 10 Word List Recall, Telephone Interview for Cognitive Status (Telephone adaptation of MMSE)	The risk of substantial cognitive decline in women with migraine or a past history of migraine was not increased compared to women without migraine	Rist et al., 2012 [28]
Unspecified^c^	Recognition memory	Longitudinal, population based	Benton Facial Recognition Test	No significant difference between migraine patients and controls	Rist et al., 2011 [27]
Unspecified	Information processing speed	Cross sectional, population based	Verbal learning test	Migraine had no influence on processing speed in young/middle-aged or older adults	Jelicic et al. 2000 [21]
Unspecified		Cross sectional, population based	Symbol Digit Substitution Task	No difference between migraineurs and non-migraineurs	Gaist et al., 2005 [22]
Unspecified^a^		Cross sectional, population based	Letter–digit substitution test	No difference between migraine patients and controls	Wen et al., 2016 [23]
Unspecified^b^		Longitudinal, population based	Stroop Test, Letter Digit Substitution Test	No difference between migraine patients and controls	Baars et al., 2010 [29]
Post-ictal, interictal	Perceptual organization	Cross sectional, clinic based	Computerized global-local test	No difference between migraine patients and controls regarding perceptual organization capabilities	Koppen et al., 2011 [17]

^a^Migraine patients were categorized as active (< 1 year since the last attack) or non-active (> 1 year since last attack) migraine

^b^Active migraine patients were included in the study

^c^Patients with a lifetime history of severe headache had a phone interview with a neurologist for migraine diagnosis

^d^Both women with migraine and women with a past history of migraine were included in the study

^e^Time since the last attack in migraine patients were reported as < 6 months since last attack, 6 months-1 year since last attack and > 1 year since last attack

*MoCA* Montreal Cognitive Assessment, *MMSE* Mini-Mental State Examination, *WMS* Wechsler Memory Scale

Migraine prophylactic medications (e.g. topiramate) or prevalent comorbidities (e.g. depression and anxiety) can also contribute to but cannot solely account for the cognitive impairment in migraineurs. For example, drug-naïve migraine without aura patients were shown to have significantly lower scores on the total Montreal Cognitive Assessment scale in 4 out of 6 cognitive subdomains (executive function, attention, visuospatial memory, and verbal memory) compared to healthy controls with similar psychological profiles [6]. Unfortunately, few studies directly examined the impact of psychiatric comorbidities [22, 26, 27, 38] and medications [14, 15, 29, 39] on cognitive function in migraineurs. Gaist et al. adjusted the difference in cognitive scores between migraine patients and controls for the effect of depression and less marked difference was found [22]. The data from the third and fourth waves of the Baltimore Epidemiologic Catchment Area study revealed that depression had no impact on cognitive function in migraine patients [26], and in the EVA study adjusting the cognitive test results for depression had no impact on the results [27]. In another study, no difference in executive function was found between migraineurs with psychiatric disorders (depression and obsessive compulsive disorder) and controls [38]. Lastly, studies on the effects of preventive medications and acute attack treatment on cognitive function in migraineurs did not find a significant impact on cognitive function [14, 15, 29, 39].

Because migraine aura is caused by cortical spreading depression, an intense neuronal and glial depolarization wave that is known to disrupt cortical function and cause lasting cerebrovascular dysfunction [40, 41], and because aura is a risk factor for white matter lesions and cerebrovascular events [42], it is worth mentioning that a small number of studies with heterogeneous methodologies have investigated cognitive function in migraineurs with or without aura. The results of these studies were mixed and non-conclusive. Although migraineurs with aura appeared to have more prominent cognitive impairment particularly with tasks evaluating sustained attention and processing speed [14, 43], and showed anomia and prosopagnosia [44, 45], other studies found no difference in cognitive performance between migraineurs with or without aura [28], and as mentioned above, some even showed better cognition in migraineurs with aura [23].

Altogether, discrepant results among studies on cognitive function in migraine can be attributed to 1) ictal versus interictal assessment, 2) clinic versus population based recruitment (e.g. sample size, diagnostic accuracy, disease severity), 3) cross-sectional versus longitudinal design, 4) differences in clinical characteristics (e.g. age, aura, pain intensity, attack frequency and duration), 5) migraine preventive treatments, and 6) comorbidities (e.g. vascular risk factors, affective disorders).

### Dysfunctional cognitive domains in migraine

Cognitive impairment in cross sectional, clinic-based studies showed that migraine affected certain cognitive domains in particular, such as processing speed, attention, memory, verbal skills and executive function (e.g. working memory, divided attention/inhibition, set-shifting, and planning). Migraine had a moderate to marked effect on processing speed and visuomotor scanning speed [14, 39, 46–48], whereas basic attention [14–16, 46, 48, 49] and delayed verbal memory [14–16, 46, 49–51] were mildly affected, and more complex psychomotor processing speed tasks were not significantly affected [16, 50]. Some studies observed mild to moderate impairments in non-verbal memory (e.g. immediate figure recall tests) [14, 15, 46] whereas others found no effect or better performance in migraineurs [49, 52]. Verbal skills (auditory comprehension, reading, aphasia screening, verbal reasoning, vocabulary, phoneme detection) were mildly impaired [14, 16, 47, 50, 51]. In terms of executive function, migraine had a moderate to marked effect on sustained attention and working memory [16, 39, 43]. There was slight dysfunction in the inhibition domain in migraine patients [16, 39, 43]. In the domains of mental flexibility and set shifting, several studies reported that migraine patients exhibited a moderate or marked impairment [14, 38, 47]. One study that included problem solving and decision making also found a marked impairment in these domains in migraine patients [38].

### Neuroimaging, neurophysiological and pharmacological studies

The predominant involvement of processing speed, sustained attention and memory suggested prefrontal and temporal cortical dysfunction during the attacks [8], also supported by functional imaging studies [53, 54]. A positron emission tomographic study showed activation of prefrontal cortex and temporal lobe during migraine attacks [53], and an fMRI study revealed significantly greater activation in the medial temporal lobe [54]. In the latter study, temporal lobe showed increased functional connectivity with several brain regions in migraineurs relative to controls in response to painful heat, and fMRI activation in temporal lobe was exacerbated during migraine headache attacks.

The functional organization of brain networks associated with pain and cognitive processes may be altered in migraine. De Tommaso et al. showed that episodic or chronic migraine patients have deficits in cognitive task-related suppression of laser evoked potential amplitudes during acute pain [55]. fMRI studies reveal blunted cognitive-related neural activity in migraine patients [56]. While healthy subjects have strong task-related deactivation in the left dorsolateral prefrontal cortex, dorsal anterior midcingulate cortex, and cerebellum that is decreased with acute pain, migraineurs show blunted task-related deactivation with no change in response to acute pain. These changes were not associated with pain catastrophizing or pain intensity.

In a fMRI study, migraine without aura patients showed aberrant intrinsic connectivity within the bilateral central executive network (CEN) and salience network (SN), and greater connectivity between the default mode network (DMN) and right CEN (rCEN) and the insula. Moreover, greater connectivity between the DMN and rCEN and the insula correlated with duration of migraine. Both the DMN and CEN are related to cognition. CEN is associated with higher-order cognitive processes, working memory and attention. DMN is involved in specific cognitive domains such as social cognition, semantic and episodic memory and future planning. A possible neurobiological mechanism underlying cognitive deficits in migraine could be pain-related reorganization of intrinsic connectivity networks [57]. In another fMRI study, the association between cognitive functions and cerebral functional connectivity (FC) in migraine without aura, migraine with aura patients and healthy controls during interictal period was investigated [20]. A battery of neuropsychological tests was used to assess cognitive functions and no significant difference was found between three groups. However, migraine with aura patients showed altered functional connectivity compared to migraine without aura patients and healthy controls. An increased connectivity in left angular gyrus, left supramarginal gyrus, right precentral gyrus, right postcentral gyrus, right insular cortex was observed in migraine with aura patients compared to migraine without aura patients.

Event related potentials recorded by electroencephalography or magnetoencephalography have been used to evaluate cognitive processing. Electroencephalographic P3 (third positive wave around 300 milliseconds), and its magnetoencephalographic counterpart (P3m), have been shown to correlate with attention, information processing and executive function. P3 latency reflects the length of the stimulus processing time and P3 amplitude changes reflect neural activity related to cognition. P3 amplitude depends on the amount of attention given to the stimulus, working memory and the complexity of the task. Migraine patients have prolonged P3 latencies indicating prolonged cognitive processing time [13, 58]. Some of the previous studies revealed decreased P3 amplitudes in migraine patients without a significant change in P3 latencies [59, 60] whereas others showed significant elongation of P3 latencies with reduced P3 amplitudes [58].

Somatosensory temporal discrimination (STD) measures the temporal threshold to perceive two separate somatosensory stimuli as clearly distinct. STD allows the brain to process information for selecting the accurate entry of each external stimuli which is crucial for the survival and to generate proper reactions. Prolonged STD threshold (STDT) values have been reported in neurodegenerative disorders such as Parkinson’s disease, multiple system atrophy, and cerebellar atrophy. In episodic migraine patients STDT values are transiently but markedly increased during migraine attacks (3-fold higher than interictal) indicating an impairment in higher cognitive processing of somatosensory stimuli [61, 62]. In contrast to episodic migraine patients in whom interictal STDTs were normal, STD prolongation was detected on both headache days and headache free intervals of chronic migraine patients [63]. Therefore persistent elevation of STDT values above 100 ms in chronic migraine could indicate unremitting cognitive problems associated with CM. The latter was also supported by clinical neuropsychological studies which revealed cognitive impairment in CM patients [38]. In summary, processing of two discrete somatosensory stimuli remained disrupted in chronic migraine brain throughout the headache and headache-free days, showing persistent impairment in cognitive sensory processing.

Short latency afferent inhibition (SAI) is the modulation of motor response by a sensory stimulus and is known to be associated with sensorimotor integration, cognitive functions and cholinergic system. In SAI paradigm, a preceding electrical stimulation of a peripheral nerve (conditioning afferent stimulus) transiently suppresses transcranial magnetic stimulation (TMS)-induced motor output. Inhibition of the motor response occurs if the interstimulus interval between the electrical stimulation and TMS is 19–50 milliseconds [64]. In cognitive disorders such as Alzheimer dementia, mild cognitive impairment and Parkinson’s disease with dementia, SAI is shown to be reduced and rivastigmin, a cholinergic drug, augments SAI [65]. Recently it is reported that SAI is reduced during a migraine attack and is normal interictally and it is probably associated with cognitive disturbances during a migraine attack [66].

Donepezil, an acetylcholinesterase inhibitor was able to induce antinociception in mice in a dose dependent manner. Donepezil induced antinociception was shown to be dependent on cholinergic activation since it was inhibited by a non-selective muscarinic antagonist scopolamine. In an open label clinical pharmacological study, donepezil was found to be effective in migraine prophylaxis both in episodic and chronic migraine patients [67]. There seems to be a cholinergic dysfunction in migraine and since cholinergic activity of the cortex is linked to cognitive functions, cholinergic dysfunction may be related to cognitive symptoms during a migraine attack.

In two small open label studies [9, 10], cognitive functions were evaluated during interictal period, untreated migraine and following an anti-migraine drug, sumatriptan administration. Cognitive impairment was observed during the migraine attack compared to interictal period and cognitive functions were shown to be restored after sumatriptan administration (6 mg subcutaneous injection or 20 mg nasal spray).

### Other primary headache disorders

Cluster headache primarily affects men and has only one-tenth the incidence of migraine, but causes disability comparable to migraine [2]. There are few studies on cognitive performance in cluster headache. Although patients display a reversible cognitive decline during the cluster attacks, their cognitive performance was detected as normal between the attacks [68, 69].

In TTH, acute headache was associated with reversibly impaired cognitive function [70]. In a longitudinal birth cohort study, childhood headache was related to worse performance on cognitive measures such as verbal and performance IQ, receptive language, and reading, whereas cognitive performance of adults with TTH was similar to headache-free controls or headache-free tinnitus sufferers [71]. TTH, known as the most common headache disorder, is often misdiagnosed in patients with probable migraine, and chronic migraine, resulting in a highly varying prevalence of TTH between 5.1–78% [1, 2]. It is likely that majority of TTH studies are not conducted on pure TTH patients and that may be at least partially responsible for cognitive problems detected in TTH studies. Indeed, STD test was intact during headache attacks in pure TTH patients [62], while STDTs were significantly elevated in migraine attacks. STD differentiates the central pathology of migraine from TTH and, normal STDTs in TTH could suggest a better cognitive status in pure TTH attacks.

## Conclusion

Standardized neuropsychological tests show that migraine attacks are associated with poor cognitive performance compared with headache-free periods, consistent with cognitive difficulties subjectively reported during attacks. Outside the attacks, most clinic-based studies found worse cognitive performance in migraineurs. Most population-based studies reported similar cognitive performance in migraineurs and controls during the interictal period. Longitudinal studies yielded no evidence of increased risk for cognitive decline in migraine patients. Prophylactic medications and comorbid disorders such as depression and anxiety have to be recognized as confounding factors. However cognitive impairment cannot be exclusively ascribed to preventive medications and psychiatric disorders. There are limited number of studies about the cognitive performance in chronic migraine patients. There are many unresolved questions and further studies are required in order to establish the extent of cognitive impairment in patients with migraine and other primary headache disorders and whether migraine prophylactic medications have an impact on reversal of cognitive dysfunction.

## Abbreviations

EVA: Epidemiology of Vascular Aging Study
fMRI: Functional Magnetic Resonance Imaging
ICHD: International Classification of Headache Disorders
STD: Somatosensory Temporal Discrimination
STDT: Somatosensory Temporal Discrimination Threshold
TTH: Tension Type Headache
